# Correlation of cholesteryl ester metabolism to pathogenesis, progression and disparities in colorectal Cancer

**DOI:** 10.1186/s12944-022-01629-7

**Published:** 2022-02-16

**Authors:** Zhirong Liu, Christian R. Gomez, Ingrid Espinoza, Thuy Phuong T. Le, Veena Shenoy, Xinchun Zhou

**Affiliations:** 1 Department of Biochemistry, Shanxi Medical University, Taiyuan, 030001 Shanxi Province China; 2 Department of Pathology, University of Mississippi Medical Center, Jackson, MS 39216 USA; 3 Department of Preventive Medicine, University of Mississippi Medical Center, Jackson, MS 39216 USA; 4 School of Medicine, University of Mississippi Medical Center, Jackson, MS 39216 USA

**Keywords:** Colorectal cancer (CRC), Cholesteryl ester (CE), Acyl CoA: cholesterol acyltransferases (ACATs), Neutral cholesterol ester hydrolase 1 (NCEH1), Lysosomal acid lipase (LAL), Racial and gender disparity

## Abstract

**Background:**

Colorectal cancer (CRC) is one of the most common cancers worldwide characterized by disparities in age, gender, race and anatomic sites. The mechanism underlying pathogenesis, progression and disparities of CRC remains unclear. This study aims to reveal the association of expression levels of enzymes related to cholesteryl ester (CE) metabolism with pathogenesis, progression and disparities of CRC.

**Methods:**

The differences in gene expression levels were analyzed for enzymes in CE synthesis (acyl CoA: cholesterol acyltransferase 1 and 2, ACAT1, and ACAT2), and in CE hydrolysis (neutral cholesterol ester hydrolase, NCEH1 and lysosomal acid lipase, LAL) on TNMplot platform between CRC and normal colorectal tissues (NCT) in a large cohort. The differences in protein expression levels for these enzymes were determined by Immunochemistry (IHC) performed on tissue microarray containing 96 pairs of CRC and benign colorectal tissues (BCT) from different patient populations. The expression level represented as IHC score of each enzyme was compared between CRC and BCT in entire population and populations stratified by race, gender and anatomic sites. Student’s t-test, Fisher exact test and ANOVA were used for data analysis. Significant *p* value was set at *P*<0.05.

**Results:**

The gene expression level of ACAT1 was significantly lower in CRC than in NCT (*P =* 2.15e-119). The gene expression level of ACAT2 was not statistically different between CRC and NCT. The gene expression level of LIPA (encoding LAL) was significantly higher in CRC than in NCT (*P* = 2.01e-14). No data was found for the gene expression level of NCEH1. The IHC score of ACAT1was significantly lower in CRC than in BCT in all studied populations and in sub site of colon, but not in that of rectum. The IHC score of ACAT2 was not statistically different between CRC and BCT. IHC score of NCEH1 was significantly higher in CRC than in BCT only in African American (AA) population. The IHC score of LAL was significantly higher in CRC than in BCT in all studied populations and in all sub sites. In addition, decreased ACAT1 in CRC significantly correlated to progression of CRC: the lower IHC score of ACAT1, the more advanced clinical stage of CRC will be.

**Conclusions:**

This study revealed that altered expression levels in enzymes related to CE metabolism highly correlate to the pathogenesis, clinical progression and disparities of CRC. The results will add revenue in elucidating mechanisms underlying progression of CRC, and shed light on seeking biomarkers and exploring therapeutic targets for CRC in a new direction.

## Introduction

Colorectal cancer (CRC) is one of the most common cancers worldwide, ranking the third in men and the second in women [1]. Clinically, CRC is disparate in age, gender, race and anatomic sites. A lifetime risk of developing colorectal cancer is about 1 in 23 (4.4%) for men and 1 in 25 (4.1%) for women [2]. African American (AA) has the highest incidence and mortality rate than Caucasian American (CA) and other ethnicities [3]. The mechanisms underlying pathogenesis, progression and disparities of CRC have not been fully elucidated, however it is believed that many genetic and epigenetic factors are involved in [4]. Among those, alteration in lipid metabolism is now realized as an important risk factor associated with pathogenesis, progression and disparities of CRC.

CE is a storage form lipid. Intracellular CE metabolism encompasses two reverse biological processes. One process is CE synthesis, in which free cholesterol and free fatty acids were esterified to CE mainly catalyzed by ACAT1 and ACAT2 [5]. Both enzymes have more than 50% identical gene sequences and similar functions in synthesis of CE [6]. Another process is CE hydrolysis, in which CEs are hydrolyzed into free cholesterols and free fatty mainly catalyzed by neutral cholesterol ester hydrolase 1 (NCEH1) and lysosomal acid lipase (LAL). NCEH1 hydrolyzes CEs in cytoplasm, whereas LAL hydrolyzes CE lysosome [7–9]. Metabolic pathways of CE are critical in maintenance of intracellular balance of free cholesterol, which is necessary component of membranous structures and precursors of important materials in all living cells [10]. To maintain rapid proliferation and progression, cancer cells greatly increase demands in free cholesterol [11], which can be obtained from uptake from extracellular environment, from intracellular synthesis *de novo*, and importantly from hydrolyzing CEs largely stored in lipid droplets of cancer cells [12]. Previous studies indicated that CE metabolism highly correlate to pathogenesis and progression of many cancers [13–15]. However, none of studies investigated alterations of enzymes involved in CE synthesis and hydrolysis simultaneously. To date, the association of enzymes related to CE metabolism with CRC has not been reported.

In this study, TNMplot platform was employed to study the differences in gene expression levels of enzymes related to CE metabolism between CRC and BCT; and IHC was performed for these enzymes on human CRC and BCT samples to determine the differences in their protein expression level between CRC and BCT in entire populations and populations further stratified by gender, race and anatomic site.

## Materials and methods

### Patients and sample collection

The Institutional Review Board (IRB) at the University of Mississippi Medical Center approved this study in 10/29/2012 (protocol #: 2012–0205). The study cohort comprised 96 patients with clinically diagnosed CRC enrolled at University of Mississippi Medical Center during March 2006 to March 2016. The written consent was obtained from patients before donation of their tissue samples. From each of 96 patients, one CRC sample and one BCT sample were selected in this study. Thus, a total of 192 samples, including 96 CRC and 96 BCT were used in IHC stain. The clinicopathological data were obtained from the pathology files. The length of follow-up period was defined as the interval between the date of operation and patient’s expiration date or between the date of operation and the last follow-up for surviving patients. None of identifiable information, such as patient’s name, dates of birth, and contact information was provided. The geographic, clinical and pathological information is listed in Table 1.

**Table 1:** Patient’s geographic and clinic information

Features	*N*
Age (mean ± SD)	96 (59.5 ± 13.4)
Gender	96
Male (%)	50 (52.1)
Female (%)	46 (47.9)
Race	96
African American (%)	56 (58.3)
Caucasian American (%)	40 (41.7)
Site	96
Colon (%)	61 (63.5)
Rectum (%)	35 (36.5)
Clinical Stage	92 *
Low Stage** (%)	34 (36.9)
High Stage** (%)	58 (63.1)
Margin	92 *
Positive (%)	18 (19.6)
Negative (%)	74 (80.4)
Lymph Node Metastasis	86***
Positive (%)	50 (58.1)
Negative (%)	36 (41.9)
Vital Status	96
Alive (%)	37 (38.5)
Dead (%)	59 (61.5)

*Lack of data on tumor clinical stage and surgical margin in 4 cases. ** Stage system used is the American Joint Committee on Cancer (AJCC) TMN system. In this study, the low stage CRC included all AJCC stage I and II, and the high stage CRC included all AJCC stage III and IV. ***Lack of data on lymph node metastasis in 10 cases

### Gene expression profile data analysis on the public platform

TNMplot (https://www.tnmplot.com/), a cancer microarray database and web-based data mining platform, aims to compare the gene expression in normal and tumor tissues in most major types of cancer [16]. Its gene array data were manually selected from NCBI-GEO (Gene Expression Omnibus). The gene expression levels of ACAT1, ACAT2, NCEH1 and LIPA (encoding LAL) were analyzed on TNMplot platform for the expression profiles of normal tissues and non-paired tumor tissues of colon.

### Tissue microarrays (TMA) construction

TMA were constructed using a Manual Tissue Arrayer 1 (Beecher instruments, Sun Prairie, WI, USA) for 96 pairs of CRC and BCT tissues as described previously [17]. Briefly, after histological features in glass slides were re-confirmed by two researchers including one pathologist, they were topographically correlated with the corresponding primary paraffin blocks. A 1 mm cylindrical core of target tissues was selected in each primary formalin fixed paraffin embedded (FFPE) block and transferred to the composite paraffin blocks to construct TMA blocks. The resulting composite TMA blocks were then heat-treated and sectioned at 5 μm in thickness for IHC study.

### Immunohistochemistry (IHC) and scoring

IHC was performed as described previously [17]. The primary antibody for ACAT1, ACAT2, NCEH1 and LAL was ab154396 (Abcam, Boston, MA, USA, 1:400), ab231544 (Abcam, Boston, MA, USA, 1:400), ab111544 (Abcam, Boston, MA, USA, 1:400) and NBP1–54155 (Novus Biologicals, Centennial, CO, USA, 1:400), respectively. The expression levels of ACAT1, ACAT2, NCEH1 and LAL were represented by final IHC scores with a score system as described previously [17]. Briefly, the final IHC score used in data analysis was calculated by multiplying area score (0 is for no cell stained, 1 for < 10% cells stained, 2 for 10–50% cells stained, and 3 for > 50% cells stained) with intensity score (0 is for no IHC signal, 1 for weak IHC signal, 2 for moderate IHC signal, and 3 for strong IHC signal). Thus, the final IHC score for each sample will be ranged 0 to 9. All scores were independently determined by two researchers. The mean IHC score was used as the cut off value to separate high and low expression level of each enzyme. The IHC scores for these enzymes were recorded and analyzed only for CRC cells and benign colorectal epithelial cells, but not for cells in lamina propria.

### Data analysis

Statistical significance was computed using Mann-Whitney or Kruskall-Wallis tests by the TNMplot platform. Data analysis was carried out in SPSS software (IBM SPSS Statistics 26 software) and Graphpad prism 8. Mean IHC scores were compared by independent samples t-test. Correlation of IHC scores among clinical stages were analyzed with ANOVA. Fisher exact test was used to analyze differences in rates between two groups. Significant *p* value was set at p<0.05.

## Results

### Differences in gene expression levels of enzymes related to CE metabolism between normal and tumor colorectal tissues on TNMplot platform

**Fig. 1:**
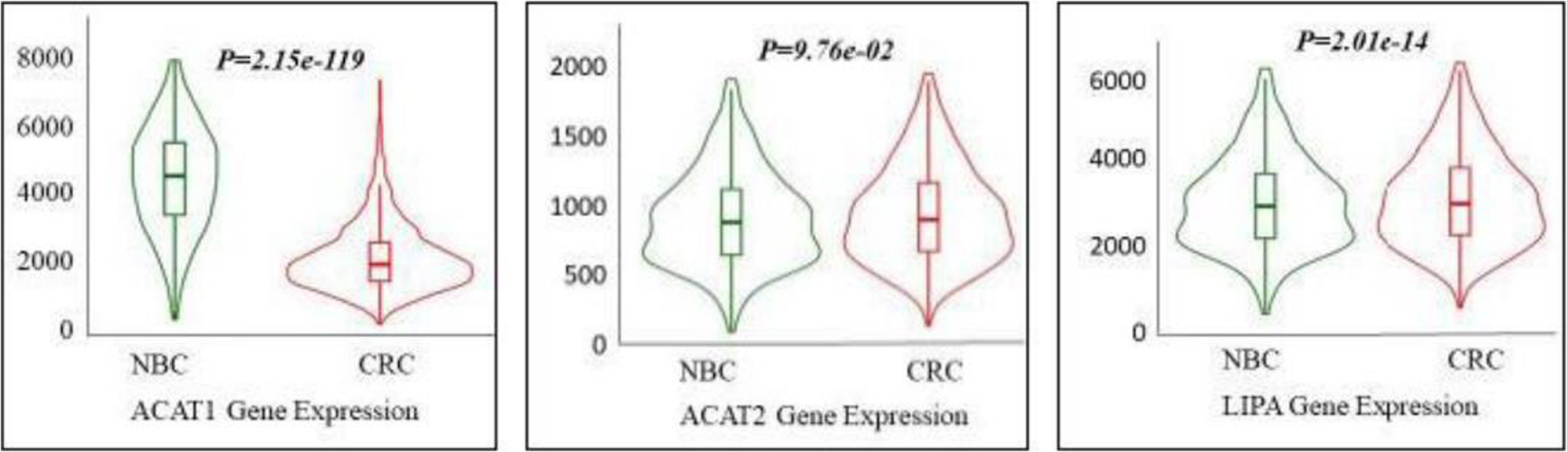
The gene expression levels of ACAT1, ACAT2 and LIPA between NCT and CRC analyzed by the TNMplot platform. **A**: The expression of ACAT1 in different tissues. **B**: The expression of ACAT2 in different tissues. **C**: The expression of LIPA in different tissues

To investigate gene expression levels of these enzymes in a larger cohort, the gene expression levels of ACAT1, ACAT2, NCEH1 and LIPA were analyzed between normal and tumor of colorectal tissues on TNMplot platform. The data of gene array were obtained from 377 normal colorectal tissues (NCT) and 1450 CRC tissues. The results shown in Fig. 1 indicated that the gene expression level of ACAT1in CRC was 0.48-fold lower than that in NCT (*P* = 2.15e-119). There was no statistical difference in the gene expression level of ACAT2 between NCT and CRC (*P* = 9.76e-02). The gene expression level of NCEH1 was not found in the platform. The gene expression of LAL in CRC was 1.19-fold higher than that in NCT (*P* = 2.01e-14).

### Differences in protein expression levels of enzymes related to CE metabolism between BCT and CRC in studied populations

**Fig. 2:**
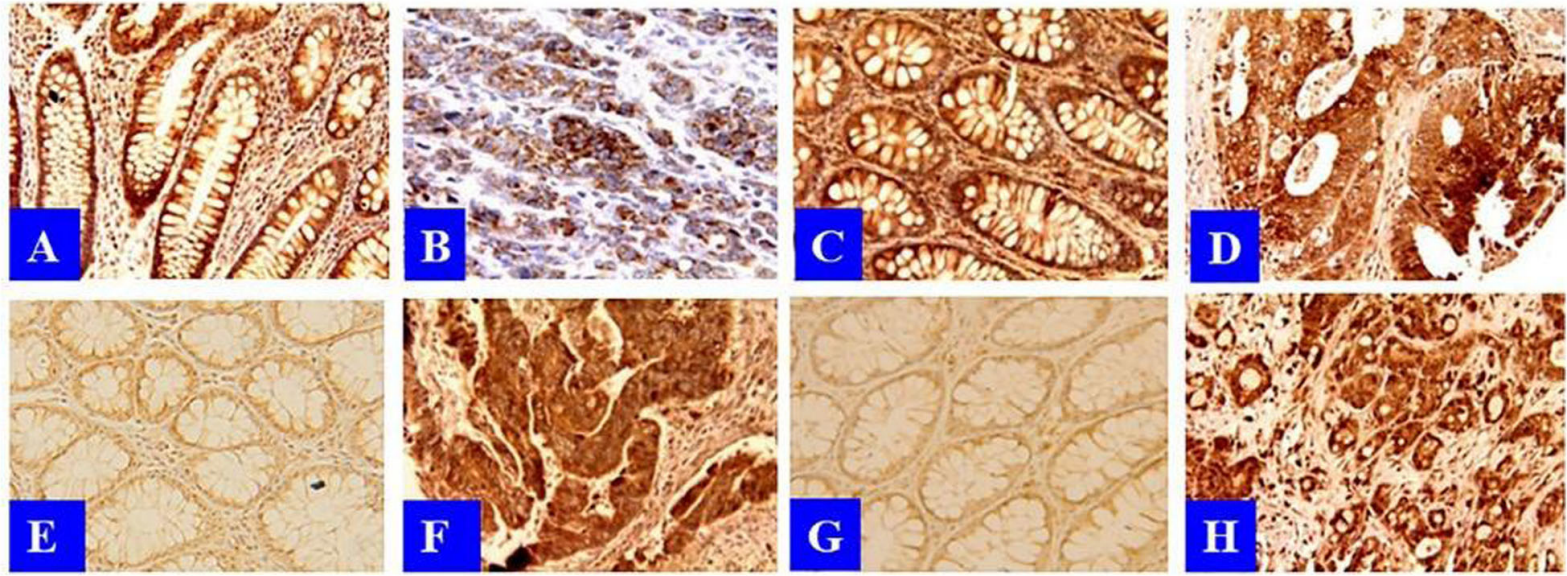
The differences in the protein expression levels of ACAT1, ACAT2, NCEH1 and LAL between BCT and CRC. A: IHC stain for ACAT1 in BCT. B: IHC stain for ACAT1 in CRC. C: IHC stain for ACAT2 in BCT. D: IHC stain for ACAT2 in CRC. E: IHC stain for NCEH1 in BCT. F: IHC stain for NCEH1 in CRC. G: IHC stain for LAL in BCT. H: IHC stain for LAL in CRC

Shown in Fig. 2 were representatives of IHC stains of one hundred twenty-two (192) BCT and CRC tissue cores for enzymes related to CE metabolism under light microscopy in high power. The IHC signals for ACAT1 were moderate in BCT (Fig. 2A) and weaker in CRC (Fig. 2B). The IHC signals for ACAT2 were moderate in both BCT (Fig. 2C) and CRC (Fig. 2D). The IHC signals for NCEH1 were weak in BCT (Fig. 2E) and moderate in CRC (Fig. 2F). The IHC signals for LAL were moderate in BCT (Fig. 2G) and strong in CRC (Fig. 2H). Overall, the IHC signals for enzymes in CE synthesis were weaker in CRC than in BCT, whereas the IHC signals for enzymes in CE hydrolysis were stronger in CRC than in BCT.

In entire population, the IHC scores for ACAT1 and LAL were significantly different between BCT and CRC: the IHC scores for ACAT1 were significantly lower in CRC than in BCT (*P* = 1.44E-7); but the IHC scores for LAL were significantly higher in CRC than in BCT (*P* = 1.81E-18). The IHC scores for ACAT2 and NCEH1 were not statistically different between BCT and CRC (Table 2).

**Table 2:** The differences in IHC scores of enzymes related to CE metabolism between BCT and CRC in entire population

Enzyme	BCT			CRC			*P* value
	*N*	Mean	SD	*N*	Mean	SD	
ACAT1	83	5.23	2.03	88	3.34	2.43	1.44E-07
ACAT2	75	5.42	2.11	83	5.49	1.86	0.815
NCEH1	83	3.83	1.92	87	4.28	1.74	0.113
LAL	83	3.74	1.71	88	6.24	1.61	1.81E-18

To analyze if the expression levels of these enzymes are disparate in gender and race, the differences in IHC scores of these enzymes were compared between BCT and CRC in stratified populations (Table 3). When entire population was stratified by gender, the expression levels of these enzymes between BCT and CRC showed similar differences in female and male populations to that in entire population (Table 3). When entire population was stratified by race, the expression levels of ACAT1, ACAT2 and LAL between BCT and CRC showed similar differences between CRC and BCT in AA population, CA populations and entire population. However, the expression level of NCEH1 significantly higher in AA CRC than in AA BCT (*P* = 0.01), whereas the expression level of NCEH1 was not statistically different between Caucasian (CA) CRC and CA BCT (*P* = 0.63). To determine if increase in expression level of NCEH1 in AA CRC was also co-influenced with genders, the expression level of NCEH1 was compared between further stratified AA female and AA male populations. In AA female population, the IHC score of NCEH1 was not significant between CRC and BCT (4.2 vs. 4.0, 1.05 fold, *P* = 0.72). In AA male population, the IHC score of NCEH1 was significantly higher in CRC than in BCT (4.8 vs. 3.3 1.46-fold, *P* = 0.00035). Thus, AA ethnicity and male gender could be cofounding factors to the elevated expression level of NCEH1 in CRC.

**Table 3:** The differences in IHC scores of enzymes related to CE metabolism between BCT and CRC in stratified populations

Population/	ACAT1	ACAT2	NCEH1	LAL
Group	N/Mean ± SD	N/Mean ± SD	N/Mean ± SD	N/Mean ± SD
Female BCT	39/5.1 ± 2.01	36/5.79 ± 1.97	40/3.83 ± 1.95	41/3.59 ± 1.68
Female CRC	42/3.35 ± 2.44	41/5.23 ± 1.84	42/4.11 ± 2.03	43/6.04 ± 1.48
*P* value	0.001	0.201	0.515	0.0000000004
Male BCT	44/5.35 ± 2.07	39/5.08 ± 2.19	43/3.84 ± 1.92	42/3.88 ± 1.74
Male CRC	46/3.34 ± 2.46	42/5.75 ± 1.87	45/4.43 ± 1.43	45/6.43 ± 1.71
*P* value	0.000068	0.14	0.10	0.0000000009
AA BCT	50/5.29 ± 1.96	44/5.32 ± 2.25	50/3.63 ± 1.77	48/3.88 ± 1.75
AA CRC	52/3.40 ± 2.35	50/5.52 ± 1.85	52/4.53 ± 1.69	49/6.37 ± 1.55
*P* value	0.000029	0.635	0.01	0.00000000005
CA BCT	33/5.15 ± 2.17	31/5.57 ± 1.91	33/4.14 ± 2.12	35/3.54 ± 1.65
CA CRC	36/3.26 ± 2.58	33/5.46 ± 1.91	35/3.91 ± 1.77	39/6.08 ± 1.68
*P* value	0.002	0.818	0.63	0.000000008

### The association of the expression levels of enzymes related to CE metabolism with progression and outcomes of CRC

**Fig. 3:**
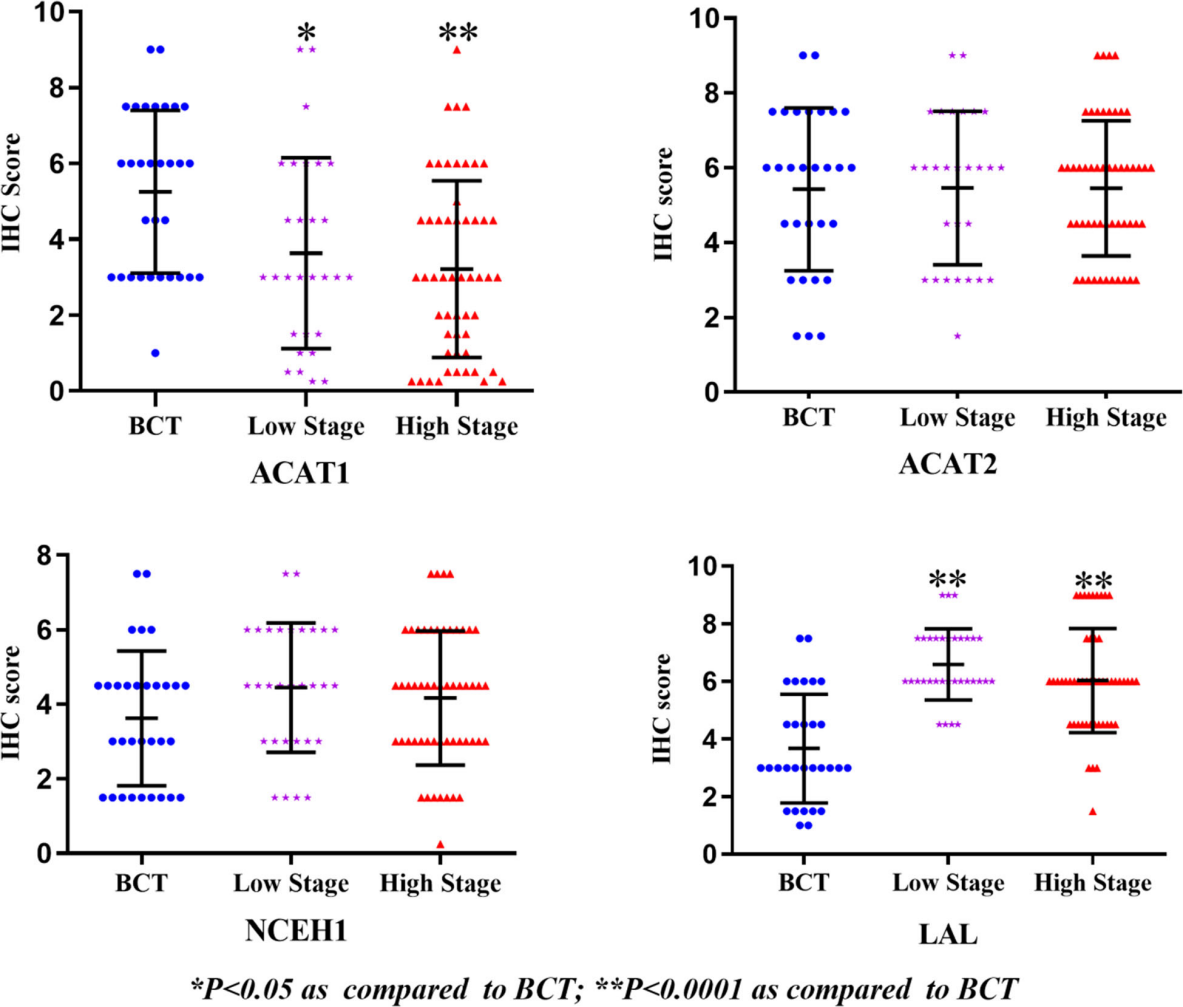
The correlation of the expression levels of enzymes related CE metabolism to clinical stages

To evaluate the associations of the expression levels of enzymes related to CE metabolism with the progression and outcomes of CRC, the difference in IHC score of each enzyme was analyzed among BCT, low stage (clinical stage I-II) CRC and high stage (clinical stage III-IV) CRC as shown in Fig. 3.

ANOVA analysis indicated that the IHC scores of ACAT1 and LAL, but not ACAT2 and NCEH1 were significantly different among groups. The IHC score of ACAT1 was highest in BCT (5.23 ± 2.03), lower in low stage CRC (3.864 ± 2.540, *P* = 0.004 as compared BCT), and lowest in high stage CRC (3.228 ± 2.174, *P* = 0.0000099 as compared to BCT. As well, the IHC score in high stage CRC was significantly lower than that in low stage CRC (3.228 ± 2.174 vs. 3.864 ± 2.540, *P* = 0.004). The IHC score of LAL were significantly higher in CRC (both low and high clinical stages) than in BCT, but there was no statistically difference in IHC score between low stage CRC and high stage CRC.

A Kaplan-Meier survival analysis was then performed to evaluate if there was a difference in the survivorship of CRC patients between high and low expression level of each enzyme (mean IHC score as cut off value). The results showed that high or low expression level of any of these enzymes did not show a significant impact on the overall survival of CRC (data not shown).

### Differences in expression levels of enzymes related to CE metabolism between BCT and CRC in different anatomic sites

CRC includes malignancy occurred in sub sites of colon and rectum. Actually, CRC is different in clinical manifestations and tumor biology between two anatomic sites. In 96 patients, 61 CRC occurred in colon, and 34 in rectum and one in both sites. Listed in Table 4 are comparison of geographic and clinical features, and expression levels of enzymes related to CE metabolism between CRCs in colon and rectum. None of geographic and clinical features was statistically different between patients with colon CRC and rectum CRC. It was noted that, however, rectum CRC was composed significantly lower percentage of AA patients than CRC in colon (47.1% in rectum CRC, 63.9% in colon CRC, *P* = 0.022).

**Table 4:** Differences in CRC features and enzyme expression levels between colon and rectum

	Geographic and clinical information					
Features	Colon			Rectum		
Cases n (%)	61 (64.2)			34 (35.8)		
% of Old (Young)	62.3 (37.7)			61.8 (38.2)		
% of Female (Male)	49.2 (50.8)			47.1 (52.9)		
% of AA (CA)*	63.9 (36.1)			47.1 (52.9)		
% of + Margin (− Margin)	13.3 (86.7)			28.1 (71.9)		
% High Stage (Low Stage)	63.9 (36.1)			60 (40)		
% of Expired (Survived)	59 (41)			65 (35)		
Survival time (month)**	46			56.4		
	Expression levels of CE metabolism related enzymes					
Enzymes	Colon			Rectum		
	BCT	CRC	*P* value	BCT	CRC	*P* value
ACAT1 (n/mean ± SD)	53/5.3 ± 1.9	60/2.9 ± 2.2	0.0001	29/5.3 ± 2.2	25/4.6 ± 2.6	0.24
ACAT2 (n/mean ± SD)	49/5.4 ± 2.1	60/2.25 ± 1.8	0.71	26/5.5 ± 2.1	22/6.2 ± 1.9	0.54
NCEH1 (n/mean ± SD)	53/4.1 ± 1.9	60/4.3 ± 1.8	0.51	29/3.5 ± 2.0	26/4.2 ± 1.7	0.14
LAL (n/mean ± SD)	53/3.8 ± 1.7	55/6.27 ± 1.8	0.0001	2.9/3.6 ± 1.8	33/6.2 ± 1.3	0.0001

* The difference is significant, P < 0.05. ** The time from diagnosis to date of expiration

The expression levels of enzymes in CE synthesis and hydrolysis were different between colon and rectum. The ACAT1 was significantly decreased in CRC only in colon, not in rectum. However, the LAL was significantly increased in CRC in both colon and rectum. Thus, less changed expression levels of enzymes in CE synthesis between BCT and CRC in rectum could be the main contributor to the difference in CE metabolism between colon and rectum.

## Discussion

Like many cancers, CRC cells require large amounts of free cholesterol to meet the demand in membrane biosynthesis and other functional needs [18]. To maintain continuous proliferation cancer cells may reprogram cholesterol metabolisms, including interaction to extracellular environment through the influx and efflux, de novo synthesis, and multiple intracellular regulatory pathways, among which, reversible conversion of free cholesterol and CE could be the most fast and cost/effective way to obtain free cholesterols. Previously, studies on changes in cholesterol metabolisms in CRC mostly focused on diet intake, systemic level, uptake by cancer cells, and de novo synthesis of free cholesterol [19]. However, there were scarcely reports on the process of intracellular conversion between free cholesterol and CE.

In this study, it was found that the gene and protein expression levels of ACAT1 were down-regulated in CRC as compared to BCT, which was in agreement with a study conducted in early1990s that ACAT activities increased in differentiated HT29 cell types, but was hardly detectable in undifferentiated cell types [20], suggesting that aggressive phenotype CRC cells related to down-regulation of ACAT. In a disagreement, another study reported that ACAT1 were highly expressed in human colorectal cancer tissues and cell lines [21]. Whether expression level of ACAT1 is decreased or increased in CRC needs further investigation. Intriguingly, increased expression level of ACAT1 also correlated to the progression of clinical stages. Thus ACAT1 could serve as biomarkers in diagnosis and prognosis of CRC, as well as therapeutic target in treatment of CRC. The results showed that the expression of ACAT2 were not significantly different in CRC and BCT in studied populations. This could be because ACAT1 and ACAT2 have different organ distribution: in physiological condition, ACAT1 is found in numerous organs and cell types, and ACAT2 is limited to liver hepatocytes and small intestinal mucosa [6, 22]. This study did not find the difference in the expression level of NCEH1 between CRC and BCT in entire population and stratified male, female and CA populations. However it was significantly higher in CRC than in BCT in AA population, especially in African American male patients with CRC. Previously, NCEH1 is more studied in macrophage and atherosclerosis [7, 23], but little is known about its roles in pathogenesis, progression disparities of CRC. Thus, this study was the first to find that NCEH1 correlates to gender and racial disparities of CRC. LAL is the only hydrolase that cleaves CEs to produce free fatty acids and free cholesterol in lysosomes [24]. Therefore, LAL plays a critical role in production of free cholesterol for rapidly proliferative cancer cells. Previous studies suggested that LAL participated in the development and progression of many cancers [11, 15, 25, 26]. However, the association of LAL with CRC has not been reported. The results in this study filled the gap with evidence that the expression of LAL in the levels of gene and protein was up-regulated in CRC.

CRC occurs in colon and rectum. Although five-year survival rates for all stages of disease are close in colon cancer (65%) and rectal cancer (66%) [27], two cancers are greatly different in many aspects [28, 29]. In addition to the differences in anatomy and functions, lipid metabolism may also contribute to the difference in tumor biology between two tumors. One study suggest that there are discrepancies in the composition of lipids such as fatty acids between colon and rectum of normal rats [30]. Another study reported that the gene of ACAT1 was downregulated in colon cancer [31]. However, none of studies compared the expressional levels of enzymes related to CE metabolism in BCT and CRC between colon and rectum. This study first compared differences in the expression levels of these enzymes between CRC in colon and rectum. The results indicated that the overall IHC score for enzymes in CE synthesis (ACAT1 + ACAT2) between rectal CRC (10.8) and rectal BCT (IHC score 10.4); however, the overall IHC score for enzymes in CE hydrolysis (NCEH1 + LAL) was higher in colon CRC (10.5) than in colon BCT (7.8). Perhaps, discrepancy in the expression level of enzymes in CE synthesis between colon BCT and rectum BCT is one of factors contributing to the difference in lipid metabolism, and in tumor biology between CRCs in colon and rectum.

Lamina propria, a layer of loose connective tissue underlying colonic mucosa contains different types of cells related to immune response in tumor microenvironment [32, 33]. The expression levels of CE metabolic enzymes in these lamina propria cells may reflect the progression of CRC, however, they are also influenced with non-cancerous conditions, such as in inflammatory bowel disease [34]. In this study, the intensity of IHC stains for enzymes related to CE metabolism in were not in concordance between lamina cells and CRC/BCT epithelia, and among the different lamina cells. Therefore, this study did not analyze the association of CRC with expression levels of enzymes related to CE metabolism in lamina cells.

### Comparisons with other studies

In address the association of cholesterol with oncogenesis, progression and disparities of CRC, previous studies focused on cholesterol metabolism in diet intake, circulatory concentration, uptake by cancer cells, and de novo synthesis. However, studies were scarce in addressing the significance of intracellular reversible conversion of free cholesterol and CE in CRC. This study filled this gap, and suggested that this intracellular process might be critical for CRC progression in production of free cholesterol in a more fast and cost/effective. In addition, this study investigated all enzymes involved in this process simultaneously. Thus, study in such way might provide more angles in observation as compared to studies addressing sole aspect in a metabolic process.

### Strengths and limitations

A strength of this study is study design, in which a meta-analysis is combined with case control to investigate correlation of enzymes related to CE metabolism with pathogenesis, progression and disparities of CRC. Other strengths is that this study may shed the light on seeking diagnostic and prognostic biomarkers, and on exploring therapeutic targets for CRC in a new direction. The limitations in study include: 1) sample size was not large enough to satisfy the power in analysis for stratified populations, 2) failed to obtain fresh frozen samples from same participants resulted in lacking transcriptional analysis and translational analysis confirmed by western blot, and 3) Incomplete laboratory data for all participants made it impossible to correlate expression of the enzymes with clinical manifestations accurately.

## Conclusions

This study revealed that altered expression levels in enzymes related to CE metabolism highly correlate to the pathogenesis, clinical progression and disparities of CRC. The results will add revenue in elucidating mechanisms underlying progression of CRC, and shed light on seeking biomarkers and exploring therapeutic targets for CRC in a new direction.

## Data Availability

The datasets during current study are available from the corresponding author on reasonable request.
